# The expression of *RUNX3* in colorectal cancer is associated with disease stage and patient outcome

**DOI:** 10.1038/sj.bjc.6604899

**Published:** 2009-02-17

**Authors:** R Soong, N Shah, B K Peh, P Y Chong, S S Ng, N Zeps, D Joseph, M Salto-Tellez, B Iacopetta, Y Ito

**Affiliations:** 1 Oncology Research Institute, Yong Loo Lin School of Medicine, National University of Singapore, Singapore, Singapore; 2 Department of Pathology, National University of Singapore, Singapore, Singapore; 3 Department of Radiation Oncology, Sir Charles Gairdner Hospital, Nedlands 6009, Australia; 4 School of Surgery, The University of Western Australia, Nedlands 6009, Australia; 5 Institute of Molecular and Cellular Biology, Proteos, Singapore, Singapore

**Keywords:** RUNX3, colorectal cancer, tissue arrays, prognosis, Wnt

## Abstract

RUNX3 is believed to have tumour suppressor properties in several cancer types. Inactivation of RUNX3 has been shown to occur by methylation-induced transcriptional silencing and by mislocalization of the protein to the cytoplasm. The aim of this study was to examine the clinical significance of RUNX3 expression in a large series of colorectal cancers using immunohistochemistry and tissue arrays. With advancing tumour stage, expression of RUNX3 in the nucleus decreased, whereas expression restricted to the cytoplasmic compartment increased. Nuclear RUNX3 expression was associated with significantly better patient survival compared to tumours in which the expression of RUNX3 was restricted to the cytoplasm (*P*=0.025). These results support a role for *RUNX3* as a tumour suppressor in colorectal cancer.

The *RUNX3* gene encodes a protein that belongs to the runt domain family of transcription factors involved in mammalian development pathways (Ito, 2008). RUNX3 protein can mediate the growth suppressive effects of TGF-*β* by associating with SMAD, a downstream protein in the signalling pathway (Ito and Miyazono, 2003). In *RUNX3* knockout mice, the gastric epithelium displays hyperplasia and a reduced sensitivity to TGF-*β* (Li *et al*, 2002). The chromosomal locus for *RUNX3* (1p36) shows frequent loss of heterozygosity in a variety of cancer types including colon and gastric carcinomas (Ito, 2008). In addition, mutations in *RUNX3* have been shown in gastric (Li *et al*, 2002) and bladder (Kim *et al*, 2005) cancers. Recent work from our group has also shown that RUNX3 protein forms a ternary complex with *β*-catenin/TCF4 (Ito *et al*, 2008). This complex has reduced DNA-binding ability and thus attenuates the level of signalling through the Wnt pathway. The above findings suggest a putative tumour suppressor role for RUNX3 in intestinal tumourigenesis.

Other studies have shown methylation-related transcriptional silencing of RUNX3 expression in gastric (Li *et al*, 2002; Waki *et al*, 2003), colorectal (CRC) (Goel *et al*, 2004; Ku *et al*, 2004) and oesophageal squamous cell (Sakakura *et al*, 2007) carcinomas. A relatively high frequency of *RUNX3* methylation has also been observed in hepatocellular carcinoma and lung, breast and prostate cancers (Kim *et al*, 2004). Mislocalisation of RUNX3 protein to the cytoplasm is another mechanism by which *RUNX3* can be inactivated in gastric and breast cancers (Ito *et al*, 2005; Lau *et al*, 2006). Overexpression of the enhancer of zeste homologue 2 (EZH2) protein was recently shown to downregulate RUNX3 expression by increasing histone H3 methylation, thus providing yet another mechanism for inactivation of *RUNX3* (Fujii *et al*, 2008). As might be expected, if RUNX3 were behaving as a tumour suppressor, the decreased expression of this protein in gastric (Wei *et al*, 2005), lung (Araki *et al*, 2005) and oesophageal (Sakakura *et al*, 2007) cancers has been associated with worse patient outcome.

As the TGF-*β* signalling pathway plays an important role in the growth control of human colonic epithelial cells (Xu and Pasche, 2007), *RUNX3* may also act as a tumour suppressor gene in this tissue. Approximately 20% of primary CRCs show hypermethylation of *RUNX3* (Goel *et al*, 2004; Ogino *et al*, 2007), and this has been linked to transcriptional silencing in two studies (Goel *et al*, 2004; Ku *et al*, 2004). The high specificity of *RUNX3* methylation in tumour tissue relative to normal colonic mucosa has led to its inclusion in a panel of five genes proposed for the standardised classification of the CpG island methylator phenotype (CIMP) in CRC (Weisenberger *et al*, 2006). Although it is clear that methylation of the *RUNX3* promoter region is one of the ways in which this gene can be inactivated in CRC, other mechanisms including mislocalisation of RUNX3 protein could also be occurring. The expression of RUNX3 in CRC as determined by immunohistochemistry has not previously been reported. In this study, we used tissue microarrays containing a very large number of primary CRC samples to investigate the expression of RUNX3 in relation to tumour features and to patient outcome.

## Materials and methods

### Tissue microarrays

Sections from tissue microarray (TMA) blocks containing 849 CRC (stages I–IV) and matching normal tissue samples were obtained from the West Australian Research Tissue Network, Department of Radiation Oncology, Sir Charles Gairdner Hospital. Construction of the TMAs and the tumour and patient characteristics have been described elsewhere (Chai *et al*, 2004). Microsatellite instability (MSI) status was determined using the BAT26 mononucleotide marker as described previously (Chai *et al*, 2004). Ethics approval to perform this study was obtained from the Human Research Ethics Committee of the Sir Charles Gairdner Hospital.

### Immunohistochemistry

Sections from tissue array blocks were cut at 5 *μ*m thickness and stained for RUNX3 by immunohistochemistry using the monoclonal antibody clone R3-6E9 as described previously by our group (Ito *et al*, 2005). The R3-6E9 clone recognises an epitope within the 191–234 amino-acid region. Its specificity for human RUNX3 has been shown by Western blot analysis and by the removal of immunoreactivity to normal gastric mucosa following preincubation with RUNX3 peptide (Ito *et al*, 2005). Rehydrated TMA sections were warmed in target retrieval solution (DAKO, Glostrup, Denmark) at 96°C for 40 min, treated with a serum-free blocking solution (DAKO), and then incubated overnight at 4°C with 1 *μ*g/ml R3-6E9 in a diluent solution (DAKO). A peroxidase-3,3′-diaminobenzidine-based detection system (EnVision+kit, DAKO) was used to detect immunoreactivity. Staining was graded for intensity on a scale of 0–4 in the nuclear and cytoplasmic compartments. The scoring was performed by a single pathologist (NS) following consultation with another pathologist (MST) and in the absence of information on patient outcome or tumour pathology. RUNX3 was considered to be expressed in the nucleus or cytoplasm if the intensity was estimated at ⩾2.

## Results and discussion

**Figure 1:**
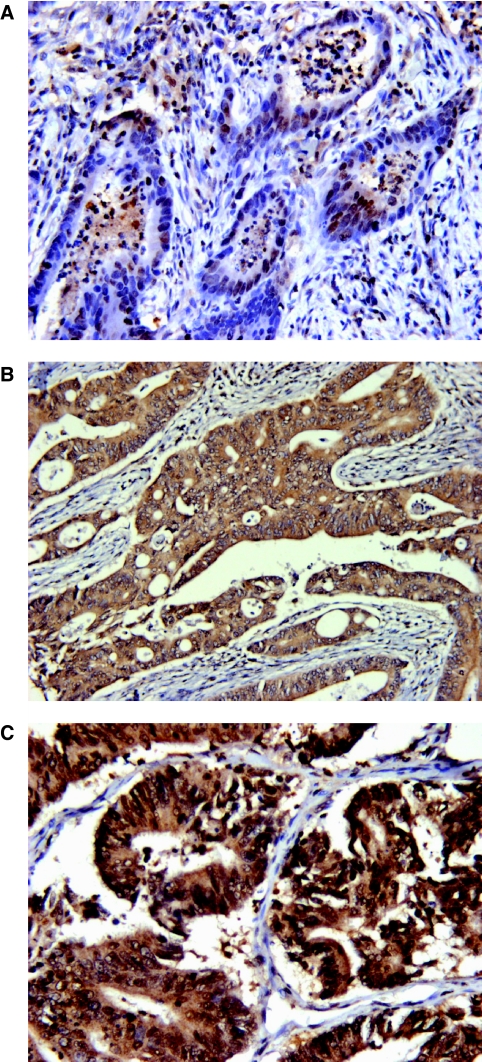
Representative images showing expression of RUNX3 in the nucleus only (**A**), in the cytoplasm only (**B**) and in both the nucleus and cytoplasm (**C**). Images are at × 40 magnification.

Typically observed immunohistochemical staining patterns for RUNX3 are shown in Figure 1. Using a staining intensity of ⩾2 as the threshold, RUNX3 expression was expressed in the nucleus of 631/849 (74%) tumours and in the cytoplasm of 431/849 (51%) tumours. Both nuclear and cytoplasmic expressions were present in 352 (41%) tumours, nuclear expression only in 279 (33%), neither nuclear nor cytoplasmic expression in 139 (16%) and cytoplasmic expression only in 79 (9%) tumours. In this study, tumours with ‘any nuclear’ staining were considered to express ‘active’ RUNX3 (*n*=631, Group A) (Lau *et al*, 2006). Tumours with no nuclear or cytoplasmic staining (Group B) or with expression in the cytoplasm only (Group C) were considered to have ‘inactive’ RUNX3.

The associations between RUNX3 expression and clinicopathological and molecular features of the CRC series are shown in Table 1. No significant associations were seen with patient age or gender, or with the histological grade of the tumour. Tumours located in the proximal colon displayed a trend for less frequent expression of nuclear RUNX3 compared to those arising in the distal colon and rectum. A likely explanation for this finding is that CIMP+ and *RUNX3* methylation are known to occur more often in the proximal colon (Weisenberger *et al*, 2006) and are associated with methylation-induced transcriptional silencing. The MSI+ phenotype is also closely associated with the CIMP+ phenotype, thus accounting for the lower frequency of nuclear RUNX3 expression observed in MSI+ tumours (Table 1).

**Figure 2:**
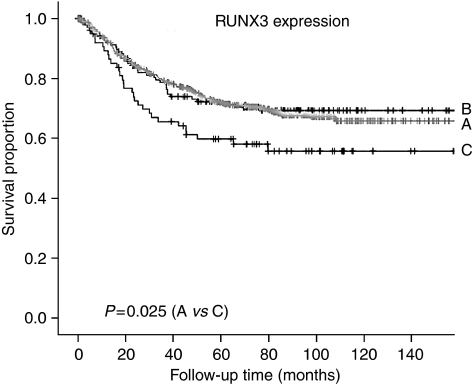
Kaplan–Meier survival analysis for CRC patients according to the expression of RUNX3. (A) Any nuclear expression (*n*=631); (B) no nuclear or cytoplasmic expression (*n*=139); (C) cytoplasmic expression only (*n*=79). Logrank test: A *vs* C, *P*=0.025.

In keeping with its postulated role as a tumour suppressor, advanced stage tumours (AJCC stages III and IV) showed less frequent expression of nuclear RUNX3 compared to early stage tumours (Table 1). Kaplan–Meier analysis confirmed that patients with nuclear RUNX3 expression (*n*=631, Group A) had significantly better survival outcomes (*P*=0.025, logrank test) compared to the relatively small number of patients (*n*=79, Group C) in which RUNX3 expression was restricted to the cytoplasm (Figure 2). This result agrees with observations made in oesophageal cancer and is consistent with current understanding of the role of RUNX3 as a tumour suppressor (Sakakura *et al*, 2007). RUNX3 expression in the cytoplasm has been reported by our group to represent mislocalised and probably inactive protein in gastric and breast cancers (Ito *et al*, 2005; Lau *et al*, 2006; Subramaniam *et al*, 2009). The present results showing that cytoplasmic expression is associated with worse patient outcome support the contention that RUNX3 is in an inactive state.

Interestingly, the subgroup of CRC patients with no or very low expression of RUNX3 in the nucleus or cytoplasm (Group B) showed similar prognosis to those with nuclear RUNX3 expression (Group A, Figure 2). This contrasts with several other cancer types (Araki *et al*, 2005; Wei *et al*, 2005; Sakakura *et al*, 2007) and suggests that the presence or absence of RUNX3 may play a lesser role to its cytoplasmic localisation in determining clinical phenotype in CRC. It should be highlighted, however, that two of the earlier studies did not distinguish between nuclear and cytoplasmic staining (Araki *et al*, 2005; Wei *et al*, 2005).

In summary, the major findings of this study were that nuclear RUNX3 expression was reduced in advanced stages of CRC and that exclusively cytoplasmic expression of RUNX3 was associated with worse patient outcome.

## Figures and Tables

**Table 1:** Associations between expression of RUNX3 and clinicopathological and molecular features of CRC

	**RUNX3 expression (%)**		
**Feature (*n*)**	**Any nuclear^a^**	**No nuclear^b^**	***P***
Total (849)	631 (74)	218 (26)	
			
*Gender*			
Male (425)	316 (74)	109 (26)	
Female (424)	315 (74)	109 (26)	NS
			
*Age*			
<71 years (429)	313 (73)	116 (27)	
⩾71 years (420)	318 (76)	102 (24)	NS
			
*Histological grade*			
Well differentiated (140)	111 (79)	29 (21)	
Moderately differentiated (343)	268 (78)	75 (22)	
Poorly differentiated (80)	62 (78)	18 (22)	NS
			
*Tumour stage*			
I and II (535)	417 (78)	118 (22)	
III and IV (313)	214 (68)	99 (32)	0.002
			
*Tumour site*			
Distal (451)	348 (77)	103 (23)	
Proximal (354)	254 (72)	100 (28)	0.079
			
*Microsatellite instability*			
Negative (716)	540 (75)	176 (25)	
Positive (87)	57 (66)	30 (34)	0.046

CRC=colorectal cancer; NS=not significant.

a Any nuclear expression is considered to represent ‘active’ RUNX3 (Group A).

b No nuclear expression is considered to represent ‘inactive’ RUNX3. This group includes tumours with no nuclear or cytoplasmic expression (*n*=139, Group B) and tumours with cytoplasmic expression only (*n*=79, Group C).
